# The genome sequence of the Common Ridge-back moth, *Epermenia chaerophyllella* (Goeze, 1783) (Lepidoptera: Epermeniidae)

**DOI:** 10.12688/wellcomeopenres.24947.1

**Published:** 2025-10-03

**Authors:** David C. Lees, Maxwell V. L. Barclay, Natural History Museum Genome Acquisition Lab, Darwin Tree of Life Barcoding Collective, Wellcome Sanger Institute Tree of Life Management, Samples and Laboratory team, Wellcome Sanger Institute Scientific Operations: Sequencing Operations, Wellcome Sanger Institute Tree of Life Core Informatics team, Tree of Life Core Informatics collective, Darwin Tree of Life Consortium

**Affiliations:** 1 Natural History Museum, London, England, UK

**Keywords:** Epermenia chaerophyllella; Common Ridge-back moth; genome sequence; chromosomal; Lepidoptera

## Abstract

We present a genome assembly from an individual male *Epermenia chaerophyllella* (Common Ridge-back moth; Arthropoda; Insecta; Lepidoptera; Epermeniidae). The genome sequence has a total length of 382.14 megabases. Most of the assembly (97.37%) is scaffolded into 31 chromosomal pseudomolecules, including the Z sex chromosome. The mitochondrial genome has also been assembled, with a length of 15.2 kilobases. This assembly was generated as part of the Darwin Tree of Life project, which produces reference genomes for eukaryotic species found in Britain and Ireland.

## Species taxonomy

Eukaryota; Opisthokonta; Metazoa; Eumetazoa; Bilateria; Protostomia; Ecdysozoa; Panarthropoda; Arthropoda; Mandibulata; Pancrustacea; Hexapoda; Insecta; Dicondylia; Pterygota; Neoptera; Endopterygota; Amphiesmenoptera; Lepidoptera; Glossata; Neolepidoptera; Heteroneura; Ditrysia; Obtectomera; Epermenioidea; Epermeniidae; *Epermenia*; *Epermenia chaerophyllella* (Goeze, 1783) (NCBI:txid1178097)

## Background

*Epermenia chaerophyllella* (Goeze, 1783), also known as the Common Ridge-back, is a moth in the family Epermeniidae, with a forewing length of 5.5–6 mm ( Sterling *et al.*, 2023) or a wingspan of approximately 12–14 mm. The wings, with a whitish, yellowish, greyish or light brownish background, are variably patterned with darker markings, sometimes including black spots and particularly a dark oblique median fascia. There are four scale tufts along the dorsum, those nearest the thorax being more prominent; the forewing apex is slightly falcate. It is most easily confused with the very localised (in the United Kingdom) *E. aequidentellus* (Hofmann, 1867), and externally it is more or less identical to *E. sinjovi* Gaedike, 1993 (sympatric in the Alps; see below).

The Common Ridge-back is at least bivoltine, flying from May to September (with peaks in mid-April and from mid-June to early July: Norfolk Moths, 2025), and overwinters as an adult, with adults therefore recorded in every month. It is crepuscular and is also attracted to light at night ( Sterling *et al.*, 2023). The whitish or greyish larva, with three rows of black pinacula on each side, feeds from May to September on Apiaceae, particularly on Hogweed *Heracleum sphondylium* L., Wild Angelica *Angelica sylvestris* L., Wild Carrot *Daucus carota* L., Cow Parsley *Anthriscus sylvestris* (L.) Hoffm., and Wild Parsnip *Pastinaca sativa* L. (see Lepiforum, 2025 for a more comprehensive list of foodplants). The larvae start off as leaf miners, later often feeding communally in a sparse silk web on the underside of the leaf ( Sterling *et al.*, 2023). The pupa, with projecting wingcases, is light brown and formed in an open network cocoon ( Norfolk Moths, 2025). The moth occurs in a wide variety of habitats, including gardens.

*E. chaerophyllella* is widespread and common in the United Kingdom ( NBN Atlas Partnership, 2025) and in Ireland ( Moths Ireland & National Biodiversity Data Centre, 2025). In the Palaearctic, the species ranges from Great Britain and Western Europe south to the Mediterranean peninsulas, Sardinia and Crete, and north to Scandinavia, while extending east to Turkey, Kyrgyzstan and the Caucasus of European Russia ( GBIF Secretariat, 2025; Lepiforum, 2025).

The DNA barcode from the mitogenome assembly (OZ173998.1) presented here represents the COI-5P cluster Barcode Index Number (BIN) BOLD:AAC9729, with a maximum variability of 1.48% ( *n* = 65). It is identical or nearly identical to most European exemplars on BOLD (accessed 04/08/2025), and up to 1.05% different from haplogroups from Scandinavia. The nearest neighbour is BOLD:ACS3942 ( *Epermenia sinjovi*, from Eastern Asia, also recorded unexpectedly from the Alps of Central Europe) at 6.49% *p*-distance (see Huemer *et al.*, 2023). *Epermenia albapunctella* (Busck, 1908), from Canada (BIN BOLD:AAF0142), is equally distant from *E. chaerophyllella* ( Huemer *et al.*, 2023).

Phylogenetic studies had previously struggled to find a robust placement for the family. *E. chaerophyllella* was one of the exemplars used in the 19-nuclear-gene phylogeny of Regier *et al.* ( Regier *et al.*, 2013, Suppl. S2), which placed the family Epermeniidae adjacent to Schreckensteiniidae and Millieriidae, without support. Heikkilä *et al.* ( Heikkilä *et al.*, 2015) placed it, again without support, adjacent to Carposinidae and Alucitidae. However, based on 2 953 loci in 1 753 orthologous genes plus COI, Mayer *et al.* ( Mayer *et al.*, 2021, Suppls.) recovered the family as sister to Alucitidae, with this clade sister to Carposinidae + Pterophoridae.

**Figure 1.**
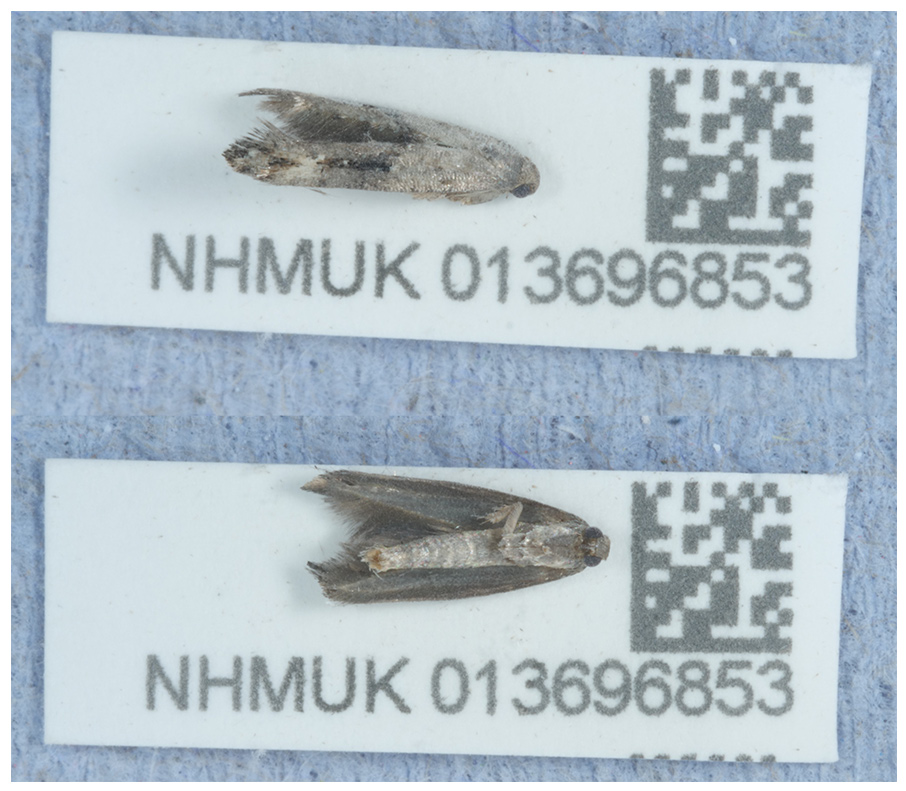
Photographs of the *Epermenia chaerophyllella* (ilEpeChae1) specimen used for genome sequencing.

This genome will add useful data towards establishing a robust phylogenetic placement and may also aid in understanding hostplant adaptations to Apiaceae secondary compounds. The assembly was produced using the Tree of Life pipeline from a specimen collected in Lucas Road, High Wycombe, England, United Kingdom ( Figure 1).

## Methods

### Sample acquisition and DNA barcoding

The specimen used for genome sequencing was an adult male *Epermenia chaerophyllella* (specimen ID NHMUK013696853, ToLID ilEpeChae1; Figure 1), collected from Lucas Road, High Wycombe, England, United Kingdom (latitude 51.63, longitude –0.74) on 2021-07-15. The specimen was collected and identified by David Lees. A second specimen was used for Hi-C sequencing (specimen ID NHMUK014584799, ToLID ilEpeChae2). It was collected from West Brompton Cemetery, England, United Kingdom (latitude 51.48, longitude –0.19) on 2022-02-14. This specimen was collected by Maxwell Barclay and identified by David Lees. For the Darwin Tree of Life sampling and metadata approach, refer to Lawniczak *et al*. (2022).

The initial identification was verified by an additional DNA barcoding process according to the framework developed by Twyford *et al.* (2024). A small sample was dissected from the specimen and stored in ethanol, while the remaining parts were shipped on dry ice to the Wellcome Sanger Institute (WSI) (see the protocol). The tissue was lysed, the COI marker region was amplified by PCR, and amplicons were sequenced and compared to the BOLD database, confirming the species identification ( Crowley *et al.*, 2023). Following whole genome sequence generation, the relevant DNA barcode region was also used alongside the initial barcoding data for sample tracking at the WSI ( Twyford *et al.*, 2024). The standard operating procedures for Darwin Tree of Life barcoding are available on protocols.io.

### Nucleic acid extraction

Protocols for high molecular weight (HMW) DNA extraction developed at the Wellcome Sanger Institute (WSI) Tree of Life Core Laboratory are available on protocols.io ( Howard *et al.*, 2025). The ilEpeChae1 sample was weighed and triaged to determine the appropriate extraction protocol. Tissue from the whole organism was homogenised by powermashing using a PowerMasher II tissue disruptor. HMW DNA was extracted using the Automated MagAttract v2 protocol. We used centrifuge-mediated fragmentation to produce DNA fragments in the 8–10 kb range, following the Covaris g-TUBE protocol for ultra-low input (ULI). Sheared DNA was purified by automated SPRI (solid-phase reversible immobilisation). The concentration of the sheared and purified DNA was assessed using a Nanodrop spectrophotometer and Qubit Fluorometer using the Qubit dsDNA High Sensitivity Assay kit. Fragment size distribution was evaluated by running the sample on the FemtoPulse system. For this sample, the final post-shearing DNA had a Qubit concentration of 2.12 ng/μL and a yield of 848.00 ng. The 260/280 spectrophotometric ratio was 1.51, and the 260/230 ratio was 1.84.

### PacBio HiFi library preparation and sequencing

Library preparation and sequencing were performed at the WSI Scientific Operations core. Prior to library preparation, the DNA was fragmented to ~10 kb. Ultra-low-input (ULI) libraries were prepared using the PacBio SMRTbell® Express Template Prep Kit 2.0 and gDNA Sample Amplification Kit. Samples were normalised to 20 ng DNA. Single-strand overhang removal, DNA damage repair, and end-repair/A-tailing were performed according to the manufacturer’s instructions, followed by adapter ligation. A 0.85× pre-PCR clean-up was carried out with Promega ProNex beads.

The DNA was evenly divided into two aliquots for dual PCR (reactions A and B), both following the manufacturer’s protocol. A 0.85× post-PCR clean-up was performed with ProNex beads. DNA concentration was measured using a Qubit Fluorometer v4.0 (Thermo Fisher Scientific) with the Qubit HS Assay Kit, and fragment size was assessed on an Agilent Femto Pulse Automated Pulsed Field CE Instrument (Agilent Technologies) using the gDNA 55 kb BAC analysis kit. PCR reactions A and B were then pooled, ensuring a total mass of ≥500 ng in 47.4 μl.

The pooled sample underwent another round of DNA damage repair, end-repair/A-tailing, and hairpin adapter ligation. A 1× clean-up was performed with ProNex beads, followed by DNA quantification using the Qubit and fragment size analysis using the Agilent Femto Pulse. Size selection was performed on the Sage Sciences PippinHT system, with target fragment size determined by Femto Pulse analysis (typically 4–9 kb). Size-selected libraries were cleaned with 1.0× ProNex beads and normalised to 2 nM before sequencing.

The sample was sequenced using the Sequel IIe system (Pacific Biosciences, California, USA). The concentration of the library loaded onto the Sequel IIe was in the range 40–135 pM. The SMRT link software, a PacBio web-based end-to-end workflow manager, was used to set-up and monitor the run, and to perform primary and secondary analysis of the data upon completion.

### Hi-C

***Sample preparation and crosslinking***

The Hi-C sample was prepared from 20–50 mg of frozen tissue of the ilEpeChae2 sample using the Arima-HiC v2 kit (Arima Genomics). Following the manufacturer’s instructions, tissue was fixed and DNA crosslinked using TC buffer to a final formaldehyde concentration of 2%. The tissue was homogenised using the Diagnocine Power Masher-II. Crosslinked DNA was digested with a restriction enzyme master mix, biotinylated, and ligated. Clean-up was performed with SPRISelect beads before library preparation. DNA concentration was measured with the Qubit Fluorometer (Thermo Fisher Scientific) and Qubit HS Assay Kit. The biotinylation percentage was estimated using the Arima-HiC v2 QC beads.

***Hi-C library preparation and sequencing***

Biotinylated DNA constructs were fragmented using a Covaris E220 sonicator and size selected to 400–600 bp using SPRISelect beads. DNA was enriched with Arima-HiC v2 kit Enrichment beads. End repair, A-tailing, and adapter ligation were carried out with the NEBNext Ultra II DNA Library Prep Kit (New England Biolabs), following a modified protocol where library preparation occurs while DNA remains bound to the Enrichment beads. Library amplification was performed using KAPA HiFi HotStart mix and a custom Unique Dual Index (UDI) barcode set (Integrated DNA Technologies). Depending on sample concentration and biotinylation percentage determined at the crosslinking stage, libraries were amplified with 10–16 PCR cycles. Post-PCR clean-up was performed with SPRISelect beads. Libraries were quantified using the AccuClear Ultra High Sensitivity dsDNA Standards Assay Kit (Biotium) and a FLUOstar Omega plate reader (BMG Labtech).

Prior to sequencing, libraries were normalised to 10 ng/μL. Normalised libraries were quantified again and equimolar and/or weighted 2.8 nM pools. Pool concentrations were checked using the Agilent 4200 TapeStation (Agilent) with High Sensitivity D500 reagents before sequencing. Sequencing was performed using paired-end 150 bp reads on the Illumina NovaSeq X.

### Genome assembly

Prior to assembly of the PacBio HiFi reads, a database of *k*-mer counts ( *k* = 31) was generated from the filtered reads using FastK. GenomeScope2 ( Ranallo-Benavidez *et al.*, 2020) was used to analyse the *k*-mer frequency distributions, providing estimates of genome size, heterozygosity, and repeat content.

The HiFi reads were assembled using Hifiasm ( Cheng *et al.*, 2021) with the --primary option. The Hi-C reads ( Rao *et al.*, 2014) were mapped to the primary contigs using bwa-mem2 ( Vasimuddin *et al.*, 2019), and the contigs were scaffolded in YaHS ( Zhou *et al.*, 2023) with the --break option for handling potential misassemblies. The scaffolded assemblies were evaluated using Gfastats ( Formenti *et al.*, 2022), BUSCO ( Manni *et al.*, 2021) and MERQURY.FK ( Rhie *et al.*, 2020).

The mitochondrial genome was assembled using MitoHiFi ( Uliano-Silva *et al.*, 2023), which runs MitoFinder ( Allio *et al.*, 2020) and uses these annotations to select the final mitochondrial contig and to ensure the general quality of the sequence.

### Assembly curation

The assembly was decontaminated using the Assembly Screen for Cobionts and Contaminants ( ASCC) pipeline. TreeVal was used to generate the flat files and maps for use in curation. Manual curation was conducted primarily in PretextView and HiGlass ( Kerpedjiev *et al.*, 2018). Scaffolds were visually inspected and corrected as described by Howe *et al*. (2021). Manual corrections included 74 breaks and 99 joins. The curation process is documented at https://gitlab.com/wtsi-grit/rapid-curation. PretextSnapshot was used to generate a Hi-C contact map of the final assembly.

### Assembly quality assessment

The Merqury.FK tool ( Rhie *et al.*, 2020) was run in a Singularity container ( Kurtzer *et al.*, 2017) to evaluate *k*-mer completeness and assembly quality for the primary and alternate haplotypes using the *k*-mer databases ( *k* = 31) computed prior to genome assembly. The analysis outputs included assembly QV scores and completeness statistics.

The genome was analysed using the BlobToolKit pipeline, a Nextflow implementation of the earlier Snakemake version ( Challis *et al.*, 2020). The pipeline aligns PacBio reads using minimap2 ( Li, 2018) and SAMtools ( Danecek *et al.*, 2021) to generate coverage tracks. It runs BUSCO ( Manni *et al.*, 2021) using lineages identified from the NCBI Taxonomy ( Schoch *et al.*, 2020). For the three domain-level lineages, BUSCO genes are aligned to the UniProt Reference Proteomes database ( Bateman *et al.*, 2023) using DIAMOND blastp ( Buchfink *et al.*, 2021). The genome is divided into chunks based on the density of BUSCO genes from the closest taxonomic lineage, and each chunk is aligned to the UniProt Reference Proteomes database with DIAMOND blastx. Sequences without hits are chunked using seqtk and aligned to the NT database with blastn ( Altschul *et al.*, 1990). The BlobToolKit suite consolidates all outputs into a blobdir for visualisation. The BlobToolKit pipeline was developed using nf-core tooling ( Ewels *et al.*, 2020) and MultiQC ( Ewels *et al.*, 2016), with containerisation through Docker ( Merkel, 2014) and Singularity ( Kurtzer *et al.*, 2017).

## Genome sequence report

### Sequence data

**Figure 2.**
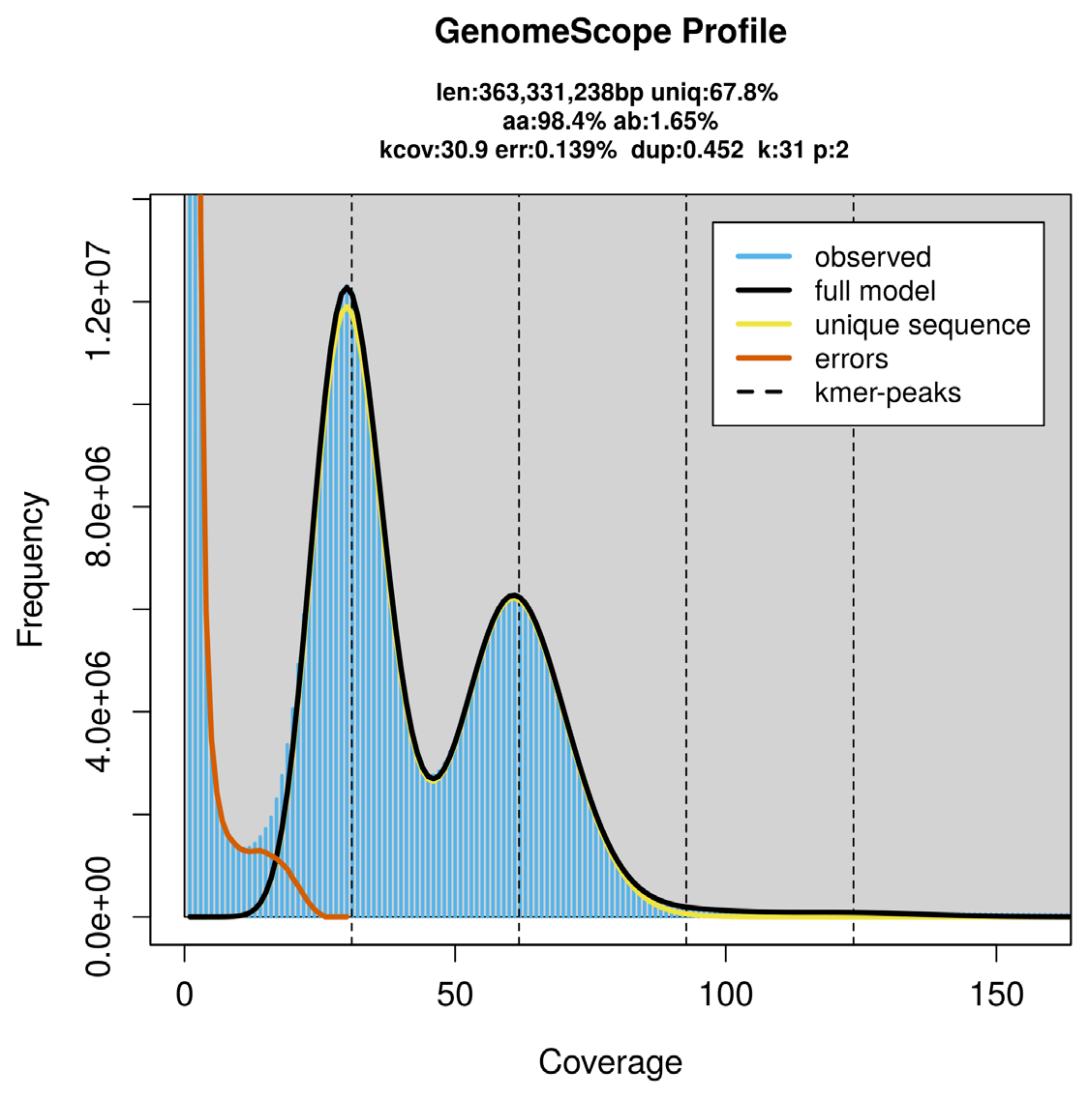
Frequency distribution of *k*-mers generated using GenomeScope2. The plot shows observed and modelled *k*-mer spectra, providing estimates of genome size, heterozygosity, and repeat content based on unassembled sequencing reads.

PacBio sequencing of the *Epermenia chaerophyllella* specimen generated 24.94 Gb (gigabases) from 2.64 million reads, which were used to assemble the genome. GenomeScope2.0 analysis estimated the haploid genome size at 363.33 Mb, with a heterozygosity of 1.65% and repeat content of 32.26% ( Figure 2). These estimates guided expectations for the assembly. Based on the estimated genome size, the sequencing data provided approximately 62× coverage. Hi-C sequencing produced 104.91 Gb from 694.74 million reads, which were used to scaffold the assembly. Table 1 summarises the specimen and sequencing details.

**Table 1.** Specimen and sequencing data for BioProject PRJEB71214.

Platform	PacBio HiFi	Hi-C
**ToLID**	ilEpeChae1	ilEpeChae2
**Specimen ID**	NHMUK013696853	NHMUK014584799
**BioSample (source individual)**	SAMEA111457881	SAMEA112221957
**BioSample (tissue)**	SAMEA111457941	SAMEA112222085
**Tissue**	whole organism	whole organism
**Instrument**	Sequel IIe	Illumina NovaSeq X
**Run accessions**	ERR12370376	ERR12742698
**Read count total**	2.64 million	694.74 million
**Base count total**	24.94 Gb	104.91 Gb

### Assembly statistics

The primary haplotype was assembled, and contigs corresponding to an alternate haplotype were also deposited in INSDC databases. The final assembly has a total length of 382.14 Mb in 209 scaffolds, with 578 gaps, and a scaffold N50 of 12.65 Mb ( Table 2).

**Table 2.** Genome assembly statistics.

**Assembly name**	ilEpeChae1.1
**Assembly accession**	GCA_964235415.1
**Alternate haplotype accession**	GCA_964235455.1
**Assembly level**	chromosome
**Span (Mb)**	382.14
**Number of chromosomes**	31
**Number of contigs**	787
**Contig N50**	1.18 Mb
**Number of scaffolds**	209
**Scaffold N50**	12.65 Mb
**Sex chromosomes**	Z
**Organelles**	Mitochondrion: 15.2 kb

**Figure 3.**
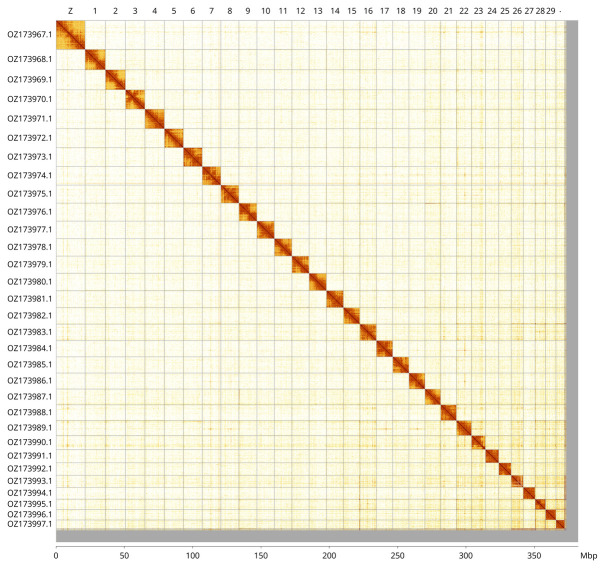
Hi-C contact map of the *Epermenia chaerophyllella* genome assembly. Assembled chromosomes are shown in order of size and labelled along the axes, with a megabase scale shown below. The plot was generated using PretextSnapshot.

Most of the assembly sequence (97.37%) was assigned to 31 chromosomal-level scaffolds, representing 30 autosomes and the Z sex chromosome. These chromosome-level scaffolds, confirmed by Hi-C data, are named according to size ( Figure 3; Table 3). Chromosome Z was assigned by synteny to the genome of *Amphipyra tragopoginis* (GCA_905220435.1) ( Boyes *et al.*, 2023).

**Table 3.** Chromosomal pseudomolecules in the primary genome assembly of *Epermenia chaerophyllella* ilEpeChae1.

INSDC accession	Molecule	Length (Mb)	GC%
OZ173968.1	1	14.94	36
OZ173969.1	2	14.68	36
OZ173970.1	3	14.23	36
OZ173971.1	4	14.22	36
OZ173972.1	5	13.96	36
OZ173973.1	6	13.76	35.50
OZ173974.1	7	13.60	35.50
OZ173975.1	8	13.25	35.50
OZ173976.1	9	13.12	36
OZ173977.1	10	12.87	35.50
OZ173978.1	11	12.72	36
OZ173979.1	12	12.69	36
OZ173980.1	13	12.65	36
OZ173981.1	14	12.43	36
OZ173982.1	15	12.15	36.50
OZ173983.1	16	11.95	36.50
OZ173984.1	17	11.93	36
OZ173985.1	18	11.87	36.50
OZ173986.1	19	11.74	36
OZ173987.1	20	11.55	36.50
OZ173988.1	21	11.54	36
OZ173989.1	22	10.99	36.50
OZ173990.1	23	10.19	36.50
OZ173991.1	24	9.73	36.50
OZ173992.1	25	9.13	37
OZ173993.1	26	8.80	37.50
OZ173994.1	27	8.67	36.50
OZ173995.1	28	7.66	37.50
OZ173996.1	29	7.65	37
OZ173997.1	30	6.15	37
OZ173967.1	Z	21.26	36

The mitochondrial genome was also assembled. This sequence is included as a contig in the multifasta file of the genome submission and as a standalone record.

**Figure 4.**
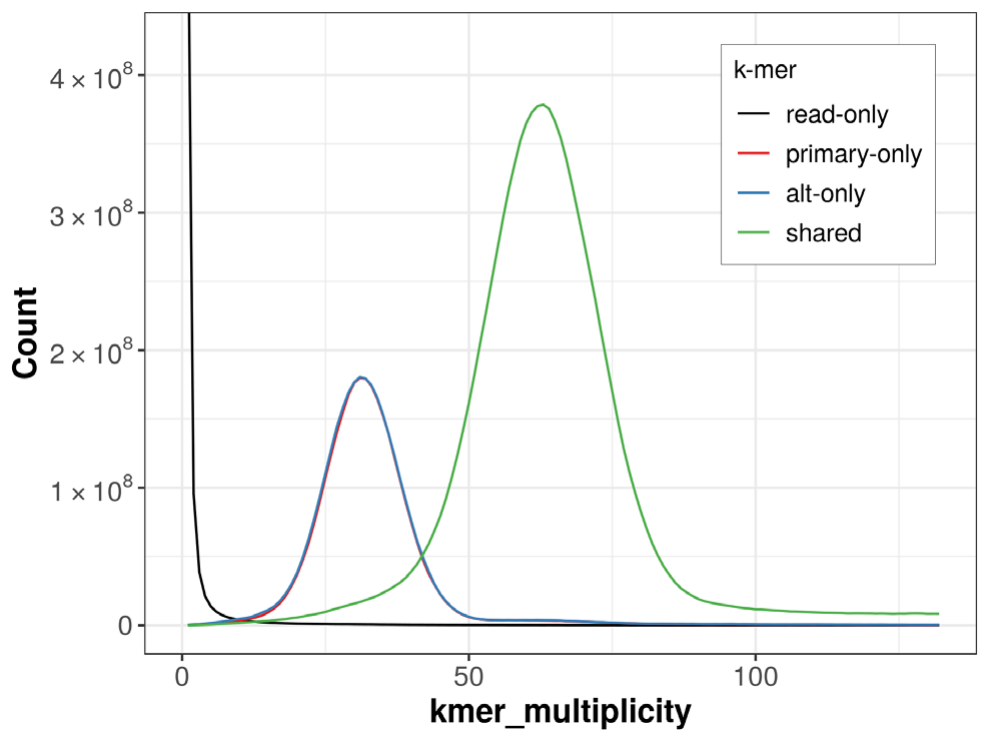
Evaluation of *k*-mer completeness using MerquryFK. This plot illustrates the recovery of *k*-mers from the original read data in the final assemblies. The horizontal axis represents *k*-mer multiplicity, and the vertical axis shows the number of *k*-mers. The black curve represents *k*-mers that appear in the reads but are not assembled. The green curve corresponds to *k*-mers shared by both haplotypes, and the red and blue curves show *k*-mers found only in one of the haplotypes.

The combined primary and alternate assemblies achieve an estimated QV of 59.7. The *k*-mer completeness is 72.64% for the primary assembly, 73.33% for the alternate haplotype, and 99.49% for the combined assemblies ( Figure 4).

**Figure 5.**
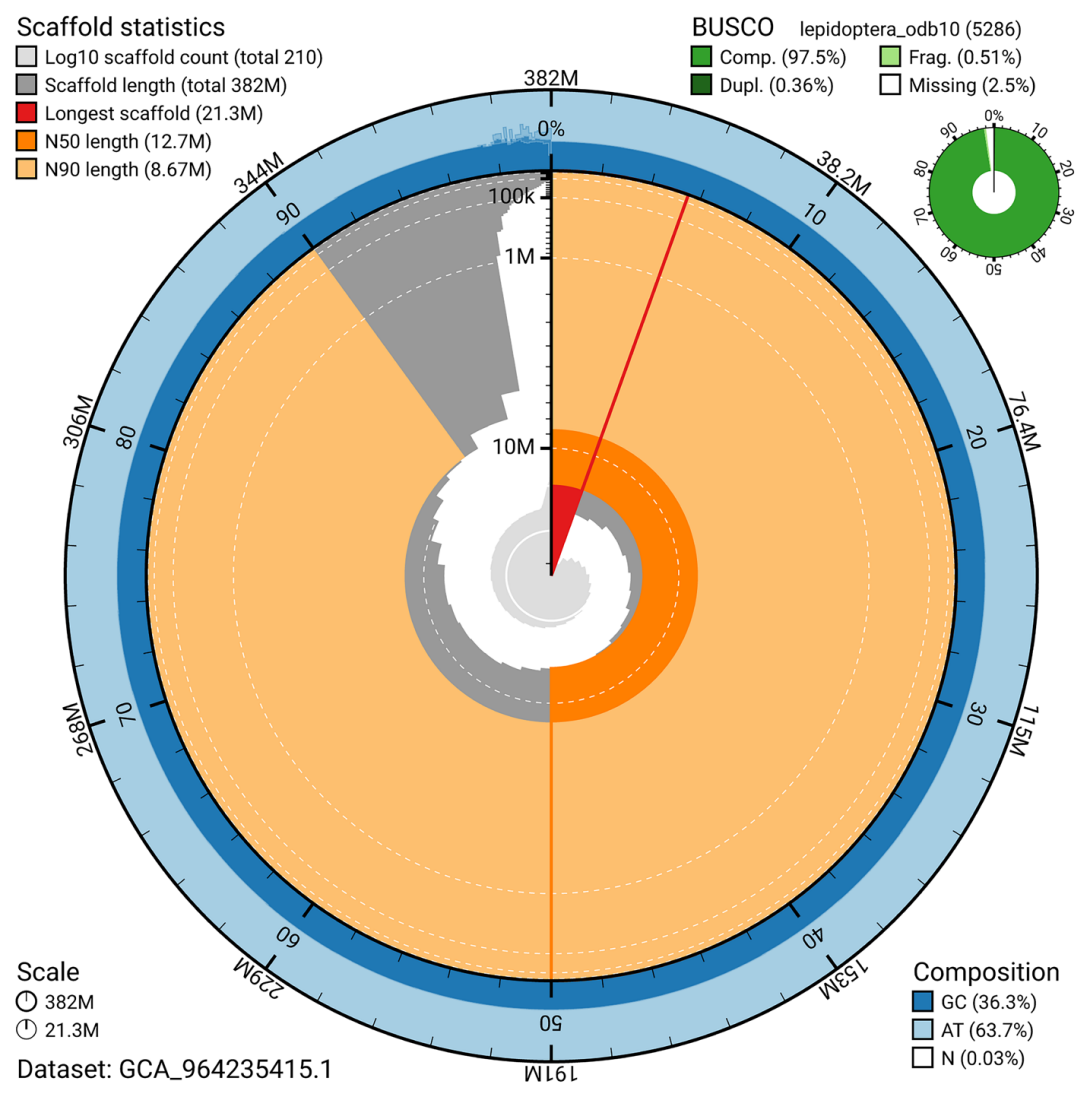
Assembly metrics for ilEpeChae1.1. The BlobToolKit snail plot provides an overview of assembly metrics and BUSCO gene completeness. The circumference represents the length of the whole genome sequence, and the main plot is divided into 1 000 bins around the circumference. The outermost blue tracks display the distribution of GC, AT, and N percentages across the bins. Scaffolds are arranged clockwise from longest to shortest and are depicted in dark grey. The longest scaffold is indicated by the red arc, and the deeper orange and pale orange arcs represent the N50 and N90 lengths. A light grey spiral at the centre shows the cumulative scaffold count on a logarithmic scale. A summary of complete, fragmented, duplicated, and missing BUSCO genes in the lepidoptera_odb10 set is presented at the top right. An interactive version of this figure can be accessed on the BlobToolKit viewer.

**Figure 6.**
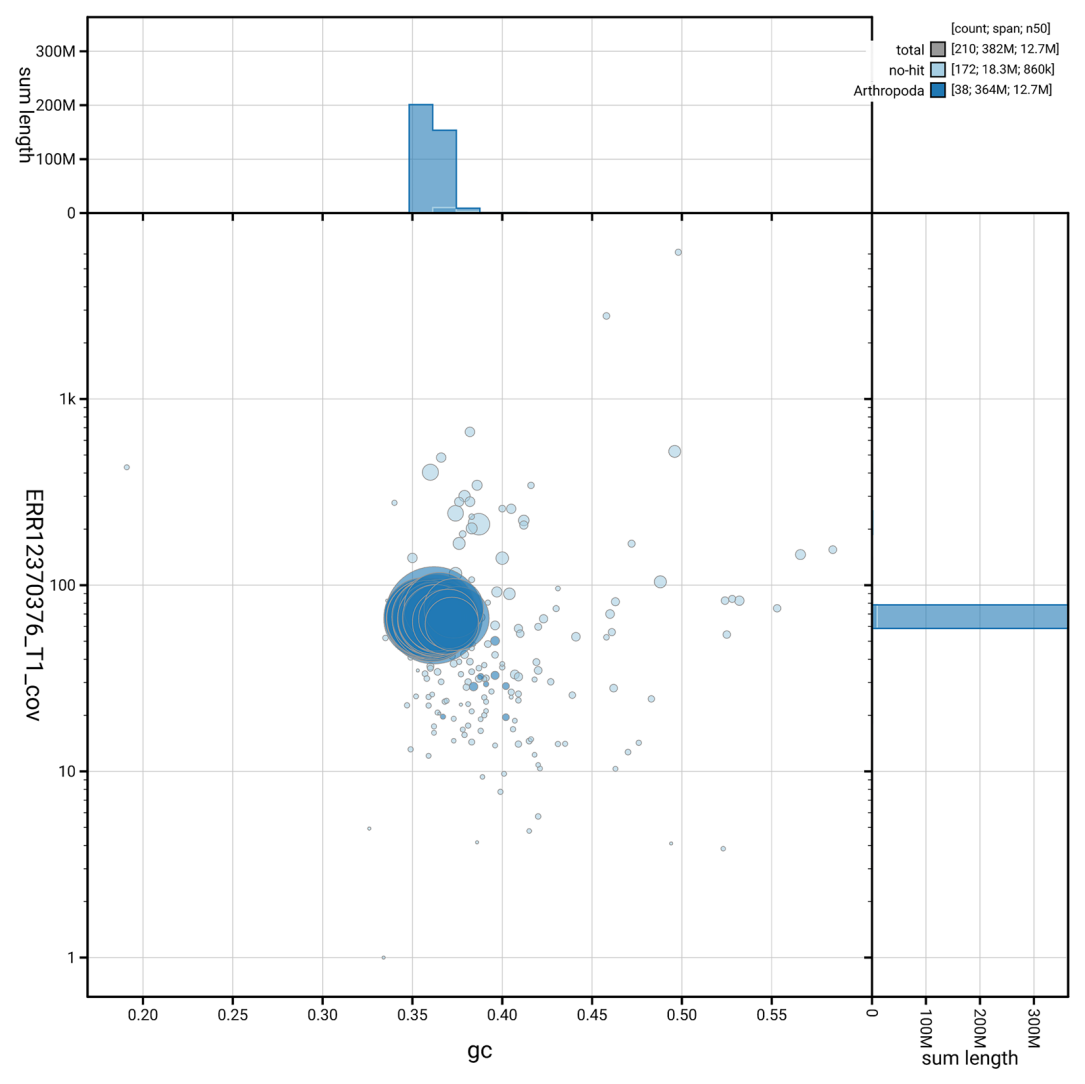
BlobToolKit GC-coverage plot for ilEpeChae1.1. Blob plot showing sequence coverage (vertical axis) and GC content (horizontal axis). The circles represent scaffolds, with the size proportional to scaffold length and the colour representing phylum membership. The histograms along the axes display the total length of sequences distributed across different levels of coverage and GC content. An interactive version of this figure is available on the BlobToolKit viewer.

BUSCO v.5.5.0 analysis using the lepidoptera_odb10 reference set ( *n* = 5 286) identified 97.5% of the expected gene set (single = 97.1%, duplicated = 0.4%). The snail plot in Figure 5 summarises the scaffold length distribution and other assembly statistics for the primary assembly. The blob plot in Figure 6 shows the distribution of scaffolds by GC proportion and coverage.

Table 4 lists the assembly metric benchmarks adapted from Rhie *et al.* (2021) the Earth BioGenome Project Report on Assembly Standards September 2024. The EBP metric, calculated for the primary assembly, is **6.C.Q58**, meeting the recommended reference standard.

**Table 4.** Earth Biogenome Project summary metrics for the *Epermenia chaerophyllella* assembly.

Measure	Value	Benchmark
EBP summary (primary)	6.C.Q58	6.C.Q40
Contig N50 length	1.18 Mb	≥ 1 Mb
Scaffold N50 length	12.65 Mb	= chromosome N50
Consensus quality (QV)	Primary: 58.8; alternate: 60.5; combined: 59.7	≥ 40
*k*-mer completeness	Primary: 72.64%; alternate: 73.33%; combined: 99.49%	≥ 95%
BUSCO	C:97.5% [S:97.1%; D:0.4%]; F:0.5%; M:2.0%; n:5 286	S > 90%; D < 5%
Percentage of assembly assigned to chromosomes	97.37%	≥ 90%

### Wellcome Sanger Institute – Legal and Governance

The materials that have contributed to this genome note have been supplied by a Darwin Tree of Life Partner. The submission of materials by a Darwin Tree of Life Partner is subject to the **‘Darwin Tree of Life Project Sampling Code of Practice’**, which can be found in full on the Darwin Tree of Life website. By agreeing with and signing up to the Sampling Code of Practice, the Darwin Tree of Life Partner agrees they will meet the legal and ethical requirements and standards set out within this document in respect of all samples acquired for, and supplied to, the Darwin Tree of Life Project. Further, the Wellcome Sanger Institute employs a process whereby due diligence is carried out proportionate to the nature of the materials themselves, and the circumstances under which they have been/are to be collected and provided for use. The purpose of this is to address and mitigate any potential legal and/or ethical implications of receipt and use of the materials as part of the research project, and to ensure that in doing so we align with best practice wherever possible. The overarching areas of consideration are:

- Ethical review of provenance and sourcing of the material
- Legality of collection, transfer and use (national and international)

Each transfer of samples is further undertaken according to a Research Collaboration Agreement or Material Transfer Agreement entered into by the Darwin Tree of Life Partner, Genome Research Limited (operating as the Wellcome Sanger Institute), and in some circumstances, other Darwin Tree of Life collaborators.

## Data Availability

European Nucleotide Archive: Epermenia chaerophyllella. Accession number PRJEB71214. The genome sequence is released openly for reuse. The *Epermenia chaerophyllella* genome sequencing initiative is part of the Darwin Tree of Life Project (PRJEB40665), the Sanger Institute Tree of Life Programme (PRJEB43745) and Project Psyche (PRJEB71705). All raw sequence data and the assembly have been deposited in INSDC databases. The genome will be annotated using available RNA-Seq data and presented through the Ensembl pipeline at the European Bioinformatics Institute. Raw data and assembly accession identifiers are reported in Table 1 and Table 2. Production code used in genome assembly at the WSI Tree of Life is available at https://github.com/sanger-tol. Table 5 lists software versions used in this study.

## Author information

Contributors are listed at the following links:

- Members of the Natural History Museum Genome Acquisition Lab
- Members of the Darwin Tree of Life Barcoding collective
- Members of the Wellcome Sanger Institute Tree of Life Management, Samples and Laboratory team
- Members of Wellcome Sanger Institute Scientific Operations – Sequencing Operations
- Members of the Wellcome Sanger Institute Tree of Life Core Informatics team
- Members of the Tree of Life Core Informatics collective
- Members of the Darwin Tree of Life Consortium
