# Supplementary material for: The genome sequence of the Common Ridge-back moth, Epermenia chaerophyllella (Goeze, 1783) (Lepidoptera: Epermeniidae)
Source: Wellcome Open Res. 2025 Oct 3;10:542. [Version 1] doi: 10.12688/wellcomeopenres.24947.1 (PMC12572775; doi:10.12688/wellcomeopenres.24947.1)
Supplement: Peer review history

# Reviewer report: Reviewer response for version 1

**DOI:** 10.21956/wellcomeopenres.27479.r135296

**Published:** 2025-10-31

**Authors:** Panagiotis Ioannidis

**Affiliations:** 1 Foundation for Research & Technology - Hellas, Crete, Greece

This manuscript reports the sequencing and assembly of the lepidopteran insect Epermenia chaerophyllella. All the genome assembly metrics show that the genome assembly is very good and suitable for downstream analyses.

 More specifically, the blobplot shows that virtually all contigs have approximately the same GC% and were sequenced at the same sequencing depth (which is amazing!). The BUSCO score is also very good as well as the QV score from Merqury.FK.

 In Table 4, I would suggest that you also add two more metrics; (1) the contig-to-chromosome (CC) ratio (Wang and Wang 2023; Trends Genet), and (2) the auN (area under the Nx curve) metric which was suggested by Heng Li:

 https://lh3.github.io/2020/04/08/a-new-metric-on-assembly-contiguity

 Lastly, I want to stress the importance of providing a high-quality gene set with each genome assembly. I noticed that the authors mention that genome annotation will take place using the "Ensembl pipeline" but this should had already been available. For non bioinformaticians a genome assembly without a gene set is of very limited use (most of the times it's the genes that you're interested in). And in order for such a gene set to be as close to reality as possible, I suggest to have as many RNAseq evidence as possible (different developmental stages and different tissues).

Are sufficient details of methods and materials provided to allow replication by others?

Yes

Is the rationale for creating the dataset(s) clearly described?

Yes

Are the datasets clearly presented in a useable and accessible format?

Yes

Are the protocols appropriate and is the work technically sound?

Yes

Reviewer Expertise:

insect genomics; bioinformatics

I confirm that I have read this submission and believe that I have an appropriate level of expertise to confirm that it is of an acceptable scientific standard.

# Reviewer report: Reviewer response for version 1

**DOI:** 10.21956/wellcomeopenres.27479.r135292

**Published:** 2025-10-29

**Authors:** Henk den Bakker

**Affiliations:** 1 University of Georgia, Griffin, Griffin, USA

This publication describes the background, rationale and methodology for sequencing the genome of a male specimen of ﻿ *Epermenia chaerophyllella. *The manuscript is generally well written, and the genome sequencing and assembly protocol is very well documented. I suggest a couple of minor revisions to improve the article:

 The species epithet suggests *Chaerophyllum temulum* is also a host plant, maybe this can be added

 …BOLD (accessed 04/08/2025)… Please write out, Barcode of Life Data Systems and include the URL

 The use of the word ‘support’ in the paragraph on phylogenetic studies in the background section; please change this into ‘phylogenetic support’, and if possible the type of support (Bayesian/bootstrap).

 In the paragraph on phylogenetic studies last sentence, replace ‘family’ with ﻿Epermeniidae.

 In the last paragraph of the background section, first sentence please change …a robust phylogenetic placement and … to …a robust phylogenetic placement of the Epermeniidae and… if that is correct.    

Are sufficient details of methods and materials provided to allow replication by others?

Yes

Is the rationale for creating the dataset(s) clearly described?

Yes

Are the datasets clearly presented in a useable and accessible format?

Yes

Are the protocols appropriate and is the work technically sound?

Yes

Reviewer Expertise:

Taxonomy, phylogenetics, genomics and bioinformatics

I confirm that I have read this submission and believe that I have an appropriate level of expertise to confirm that it is of an acceptable scientific standard.

# Reviewer report: Reviewer response for version 1

**DOI:** 10.21956/wellcomeopenres.27479.r135288

**Published:** 2025-10-09

**Authors:** Kay Lucek

**Affiliations:** 1 University of Neuchâtel, Neuchâtel, Switzerland

The authors present the chromosome level genome assembly of a male specimen of the Common Ridge-back moth *Epermenia chaerophyllella*. The assembly consists of 31 chromosomes and is not annotated for genes. The assembly is highly complete as revealed by the high BUSCO score and the Earth Biogenome Project summary metrics. Sequencing and genome assembly follow the current state of the art and use established methods. The assembly has been generated using the established pipelines of the Darwin Tree of Life Consortium.

 This is one of the best genome notes that I reviewed so far. The biological background sections is nicely elaborated and provides interesting avenues for the further use of the reference genome including taxonomy and biogeography. The methods section is moreover more detailed than many other genome notes, providing for instance, the Qubit concentrations and Nanodrop ratios, which is very helpful for other people trying to generate lepidopteran reference genomes.

Are sufficient details of methods and materials provided to allow replication by others?

Yes

Is the rationale for creating the dataset(s) clearly described?

Yes

Are the datasets clearly presented in a useable and accessible format?

Yes

Are the protocols appropriate and is the work technically sound?

Yes

Reviewer Expertise:

Speciation genomics

I confirm that I have read this submission and believe that I have an appropriate level of expertise to confirm that it is of an acceptable scientific standard.
